# Adiposity-Related Predictors of Vascular Aging From a Life Course Perspective–Findings From the Helsinki Birth Cohort Study

**DOI:** 10.3389/fcvm.2022.865544

**Published:** 2022-04-14

**Authors:** Johan G. Eriksson, Minna K. Salonen, Mikaela B. von Bonsdorff, Niko Wasenius, Eero Kajantie, Hannu Kautiainen, Tuija M. Mikkola

**Affiliations:** ^1^Department of General Practice and Primary Health Care, University of Helsinki and Helsinki University Hospital, Helsinki, Finland; ^2^Folkhälsan Research Center, Helsinki, Finland; ^3^Department of Obstetrics and Gynecology and Human Translational Research Programme, Yong Loo Lin School of Medicine, National University of Singapore, Singapore, Singapore; ^4^Singapore Institute for Clinical Sciences (SICS), Agency for Science, Technology and Research (A*STAR), Singapore, Singapore; ^5^Department of Public Health Solutions, Public Health Promotion Unit, National Institute for Health and Welfare, Helsinki, Finland; ^6^Gerontology Research Center and Faculty of Sport and Health Sciences, University of Jyväskylä, Jyväskylä, Finland; ^7^PEDEGO Research Unit, MRC Oulu, Oulu University Hospital and University of Oulu, Oulu, Finland; ^8^Department of Clinical and Molecular Medicine, Norwegian University of Science and Technology, Trondheim, Norway; ^9^Institute of Public Health and Clinical Nutrition, University of Eastern Finland, Kuopio, Finland; ^10^Clinicum, Faculty of Medicine, University of Helsinki, Helsinki, Finland

**Keywords:** vascular aging, blood pressure, body composition, pulse wave velocity, lean body mass, body fat percentage, prenatal programming

## Abstract

The main objective of this study was to study predictors of vascular health with focus on adiposity-related factors. Glucose metabolism, blood lipids, inflammatory markers and body composition were assessed 15 years before assessment of vascular health which was assessed with pulse wave velocity (PWV) in 660 subjects born 1934–44. In a univariate analysis in women the strongest association with PWV was seen for age, systolic blood pressure, dysglycemia, dyslipidemia, inflammatory markers and body fat percentage measured in late midlife and PWV measured 15 years later. In men age, body mass index (BMI), systolic blood pressure, dysglycemia, and body fat percentage in late midlife were associated with PWV. One novel finding was that adiposity-related factors were strong predictors of vascular health, something not fully encapsulated in BMI, lean body mass or body fat percentage alone. A higher fat mass index was associated with worse vascular health, which was not ameliorated by a higher lean mass index. Our findings stress the importance to study body composition and fat and lean body mass simultaneously because of their close interaction with each other also in relation to vascular health.

## What is Known About Topic

- Arterial stiffness is closely linked with blood pressure and arteriosclerosis.
- Other factors that contribute to arterial stiffness include impaired glucose regulation, low grade chronic inflammation and oxidative stress which are associated with adiposity. However, the overall influence of adiposity and body composition on vascular health has received little interest.

## What This Study Adds

- Adiposity-related factors were strong predictors of vascular health, something not encapsulated in BMI, lean body mass or body fat percentage alone. A higher fat mass index was associated with worse vascular health, which was not ameliorated by a higher lean mass index.
- The study findings stress the importance to study body composition and fat and lean body mass simultaneously also in relation to vascular health.

## Introduction

With increasing age cardiovascular disease (CVD) morbidity and mortality increase (1). Arteriosclerosis and atherosclerosis are major underlying factors influencing CVD morbidity. Both aging and hypertension are major CVD risk factors associated with the arteriosclerotic process and strongly linked to overall vascular health (2, 3). One of the core components in vascular health is believed to be arterial stiffness which is closely linked with blood pressure and arteriosclerosis (4–8).

Other factors that contribute to arterial stiffness include impaired glucose regulation, low grade chronic inflammation and oxidative stress (9–11). Several of these factors are associated with overweight and obesity and an unfavorable body composition. However, the overall influence of adiposity and body composition on vascular health has received little interest.

There is a large individual variation in the overall aging process, including vascular aging (12). This could partly be explained by factors that are active early in life; consequently, the importance to focus upon health and disease from a life course perspective has been stressed (13). Non-communicable chronic diseases (NCDs) including CVD and type 2 diabetes are known to have risk factors originating early in life, as a consequence of what is often called “early life programming” (14, 15).

The main aim of the present study was to study adiposity-related predictors of vascular health by using unique longitudinal phenotypic data from participants in the Helsinki Birth Cohort Study (HBCS) available from prenatal life until the ninth decade of life.

## Methods

Helsinki Birth Cohort Study (HBCS) consists of an epidemiological study cohort including men and women born at Helsinki University Hospital and Helsinki City Maternity Hospital between 1934 and 1944 and who attended child welfare clinics in the city, the majority also attended schools in the city of Helsinki. Details of the birth records, child welfare clinics and school health records have been described previously (14). From the epidemiological cohort we identified 8,760 people (4,630 men and 4,130 women) who were born at Helsinki University Hospital and who were living in Finland in 1971, when a unique identification number was allocated to each member of the Finnish population.

For a clinical study in the years 2001-04 we used random-number tables to select a subset of people from the initial study group who were still alive and living in Finland. In order to achieve a sample size in excess of 2,000 people for this clinical study, we selected 2,902 subjects for evaluation. Of these subjects, 2,003 attended a clinic after an overnight fast. A 2-hr 75 g oral glucose tolerance test (OGTT) was performed and blood samples were drawn for laboratory assessment including plasma glucose, insulin, blood lipids and inflammatory markers. Plasma glucose concentrations were measured according to a hexokinase method, whereas plasma insulin concentrations were determined by two-site immunometric assay (16, 17). Serum total cholesterol and triglyceride concentrations were measured with the use of standard enzymatic methods (18, 19). Height was measured with a Kawi stadiometer. Weight was measured on a Seca Alpha 770 scale. Blood pressure was measured from the right arm while the subject was in the sitting position and was recorded as the mean of two successive readings from a standard sphygmomanometer. Body composition was assessed at baseline by bioelectrical impedance analysis using the InBody 3.0 eight-polar tactile electrode system (Biospace Co, Ltd, Seoul, Korea) (20). The instrument estimates lean body mass and body fat mass by segmental multi-frequency (5, 50, 250, and 500 kHz) analysis. The measurements were made with the subject standing in light indoor clothing on the four foot electrodes on the platform of the analyzer and gripping the two palm and thumb electrodes.

Fat and lean mass indices were calculated as follows: fat mass index (FMI, kg/m^2^) = fat mass/height^2^ and lean mass index (LMI, kg/m^2^) = lean mass/height^2^ (21). We divided the study cohort based upon sex-specific median values for fat and lean mass indices. Four groups were created separately for women and men; those are: (A) low LMI and low FMI, (B) high LMI and low FMI, (C) low LMI and high FMI and (D) high LMI and high FMI.

Leisure-time physical activity (LTPA) was assessed by using a validated Kuopio Ischaemic Heart Disease Risk Factor (KIHD) Study 12-month LTPA history questionnaire (22). The subjects were asked to fill in frequency (occasions per month), average duration and intensity of each type of activity performed during the previous 12 months. For each type of activity we signed a metabolic equivalent of task (MET) -value based on available databases. To calculate the volume of LTPA in MET-hours (METh), MET values were multiplied with the average duration and frequency of activities per week.

From the original clinical study cohort (*n* = 2,003), 1,404 people who were alive and living within 100 km distance from our study clinic in Helsinki were invited to participate in a clinical follow-up in 2011. A total of 1,094 participants attended the clinical examination between 2011 and 2013. Those participants alive and living within 100 km distance from our study clinic were invited to a clinical follow-up in 2017-18. Eight hundred and fifteen people from the original clinical study cohort took part in a study to assess vascular health with pulse wave velocity (PWV) measurements (23). Of these 660 participants had valid data on both PWV and body composition and were included in the study. PWV was measured between the carotid and femoral artery with mechanotransducer sensors using the Complior (ALAM Medical, France) at rest and in supine position. The following equation was used: PWV = 0.8 × (direct distance between a. carotis and a. fermoralis measuring site/Δ time between upstroke of pressure waves). A scaling factor of 0.8 was used because direct distance leads to overestimation of real PWV (24). Two recordings were performed in supine position.

Written informed consent was obtained from each subject participating before any procedures were carried out. The Coordinating Ethics Committee, Hospital District of Helsinki and Uusimaa approved the study.

## Statistics

Data are presented as means with standard deviation (SD) or as counts (n) with percentages (%). Statistical significances for linearity across categories of carotid-femoral pulse wave velocity (CFPWV) and characteristics of the study participants were evaluated by using the Cochran–Armitage test for trend and analysis of variance (ANOVA) (with an appropriate contrast) (orthogonal). CFPWV values were divided into sex-specific four level categories (ordinals) corresponding to 20, 50, and 80th percentiles of the total distribution. The crude and adjusted relationship between body composition grouping and CFPWV were analyzed by using general linear models. In the case of violation of the assumptions (e.g., non-normality), a bootstrap-type test was used. Correlation coefficients were calculated by the Pearson method. The normality of variables was evaluated graphically and by using the Shapiro–Wilk W test. Stata 16.1 (StataCorp LP; College Station, Texas, USA) statistical package was used for the analysis.

## Results

Table 1 shows baseline characteristics in 2001-04 of the study participants at a mean age of 61 years grouped into percentiles according to PWV measured 15 years later in 2017-18, separately for women and men. Mean age of the participants in 2017-18 was 75.8 (SD 2.7) and 75.6 (SD 2.4) years in women and men, respectively. Mean BMI at follow-up was 25.8 (SD 3.1) and 26.6 (SD 4.3) kg/m^2^, respectively. Follow-up time was 14.7 (SD 0.9) years (range 12.5–16.8 years).

**Table 1:** Baseline clinical characteristics of the study participants divided into four groups of carotid-femoral pulse wave velocity (CFPWV)^*^ in the 15-year follow-up.

	**I**	**II**	**III**	**IV**	***P* for linearity**
Women, *n*	69	107	112	72	
CFPWV (m/s)	<10.05	10.05–12.00	12.05–14.50	>14.50	
Age (years)	61 (3)	61 (3)	61 (3)	62 (3)	0.016
Height (cm)	163 (5)	164 (6)	163 (5)	164 (6)	0.62
Years of education	13.2 (3.6)	12.7 (3.5)	12.4 (3.4)	12.3 (3.6)	0.093
**BP (mmHg)**
Systolic	134 (19)	139 (17)	144 (18)	145 (25)	<0.001
Diastolic	84 (9)	87 (9)	88 (10)	87 (11)	0.12
MAP	101 (11)	104 (11)	106 (11)	106 (14)	0.003
Pulse rate (bpm)	66 (9)	69 (10)	69 (10)	72 (11)	<0.001
**P-glucose (mmol/l)**
Fasting	5.35 (0.83)	5.29 (0.47)	5.51 (1.06)	5.56 (0.96)	0.041
2-hr	6.39 (2.40)	6.61 (1.60)	7.52 (3.31)	7.65 (2.16)	<0.001
Cholesterol (mmol/l)	5.86 (1.11)	5.83 (0.96)	6.07 (1.01)	6.13 (1.24)	0.047
LDL-cholesterol (mmol/l)	3.54 (0.93)	3.48 (0.84)	3.69 (0.87)	3.69 (1.07)	0.13
HDL-cholesterol (mmol/l)	1.82 (0.44)	1.77 (0.44)	1.77 (0.47)	1.72 (0.37)	0.19
Triglycerides (mmol/l)	1.12 (0.48)	1.30 (0.60)	1.40 (0.77)	1.57 (0.91)	<0.001
BMI (kg/m^2^)	25.7 (3.0)	26.8 (4.4)	27.0 (4.9)	26.4 (3.3)	0.30
LBM (kg)	47.1 (4.5)	48.0 (5.5)	47.7 (5.5)	47.1 (5.0)	0.84
Fat percentage (%)	30.2 (6.2)	32.7 (6.6)	32.6 (6.4)	33.1 (5.4)	0.014
LMI	17.7 (1.2)	17.7 (1.5)	17.9 (1.7)	17.5 (1.4)	0.83
FMI	7.9 (2.4)	9.0 (3.2)	9.0 (3.5)	8.9 (2.4)	0.073
hsCRP (mmol/l)	1.9 (1.9)	2.7 (3.0)	2.8 (3.2)	2.8 (3.4)	0.045
TNF-alfa (pg/ml)	7 (5)	19 (47)	11 (13)	18 (43)	0.27
Smoking history	25 (36)	49 (46)	40 (36)	24 (33)	0.36
METh/week	39 (26)	40 (25)	41 (28)	34 (24)	0.38
Use of lipid-lowering drugs	7 (10)	8 (7)	20 (18)	10 (14)	0.13
Use of BP-lowering drugs	18 (26)	22 (21)	27 (24)	22 (31)	0.42
Use of diabetes drugs	0 (0)	0 (0)	4 (4)	3 (4)	0.017
Men, *n*	57	88	92	63	
CFPWV (m/s)	<10.05	10.05–11.80	11.85–14.80	>14.85	
Age (years)	60 (2)	61 (2)	61 (3)	62 (3)	0.020
Height (cm)	177 (6)	177 (6)	177 (6)	178 (6)	0.59
Years of education	12.9 (3.9)	14.0 (4.0)	13.3 (3.8)	13.4 (3.7)	0.83
**BP (mmHg)**
Systolic	137 (21)	141 (16)	145 (18)	145 (17)	0.003
Diastolic	87 (11)	89 (9)	89 (10)	89 (10)	0.21
MAP	103 (13)	106 (10)	108 (11)	108 (11)	0.024
Pulse rate (bpm)	66 (11)	64 (11)	66 (10)	68 (11)	0.23
**P-glucose (mmol/l)**
Fasting	5.51 (0.57)	5.69 (0.61)	5.92 (1.26)	5.95 (0.79)	0.002
2-hr	7.35 (2.91)	6.79 (2.02)	7.75 (3.82)	7.57 (2.60)	0.23
Cholesterol (mmol/l)	4.84 (1.03)	4.72 (1.12)	4.66 (1.09)	4.85 (1.03)	0.95
LDL-cholesterol (mmol/l)	3.75 (0.71)	3.72 (0.80)	3.62 (0.90)	3.62 (0.86)	0.28
HDL-cholesterol (mmol/l)	1.47 (0.34)	1.45 (0.35)	1.42 (0.37)	1.45 (0.32)	0.56
Triglycerides (mmol/l)	1.14 (0.42)	1.22 (0.49)	1.22 (0.86)	1.25 (0.44)	0.34
BMI (kg/m^2^)	26.3 (3.2)	25.9 (2.8)	26.6 (3.2)	27.3 (2.8)	0.027
LBM (kg)	65.4 (8.4)	63.5 (5.9)	64.8 (6.2)	66.0 (7.5)	0.38
Fat percentage (%)	20.7 (4.6)	21.0 (4.7)	21.8 (5.6)	23.3 (4.4)	0.003
LMI	20.7 (1.7)	20.4 (1.3)	20.6 (1.4)	20.9 (1.6)	0.42
FMI	5.5 (1.9)	5.6 (1.7)	5.9 (2.2)	6.4 (1.8)	0.005
hsCRP (mmol/l)	1.7 (2.4)	1.6 (2.2)	2.2 (3.0)	2.1 (2.5)	0.21
TNF-alfa (pg/ml)	14 (25)	18 (49)	24 (60)	13 (23)	0.71
Smoking history	34 (60)	67 (76)	61 (66)	47 (75)	0.28
METh/week	36 (27)	38 (27)	38 (22)	36 (22)	0.98
Use of lipid-lowering drugs	8 (14)	19 (22)	16 (17)	14 (22)	0.44
Use of BP-lowering drugs	10 (18)	29 (33)	25 (27)	17 (27)	0.48
Use of diabetes drugs	2 (4)	0 (0)	4 (4)	2 (3)	0.55

*Values are given as mean and SD or proportions and percentages. Values are mean and SD or n and %. BMI, body mass index; BP, blood pressure; MAP, mean arterial blood pressure; LBM, lean body mass; LMI, lean mass index; FMI, fat mass index; CFPWV, carotid-femoral pulse wave velocity; METh, metabolic equivalent hours*.

* *CFPWV sex-specific categories corresponding to 20, 50, and 80th percentiles of total distribution*.

A univariate analysis was performed in order to identify variables with the strongest association with PWV. In women age, systolic blood pressure and mean arterial pressure, fasting and 2-h plasma glucose, total cholesterol, triglycerides, body fat percentage, pulse rate and hsCRP all measured in late midlife were associated with PWV measured 15 years later.

In men age, body mass index (BMI), systolic blood pressure, mean arterial pressure, fasting plasma glucose, body fat percentage and fat mass index in late midlife were associated with PWV measured 15 years later.

LTPA, smoking and lean body mass were not associated with PWV, nor was the inflammatory marker TNF-alfa. Neither birth weight nor BMI at 7 years of age were associated with PWV.

**Figure 1:**
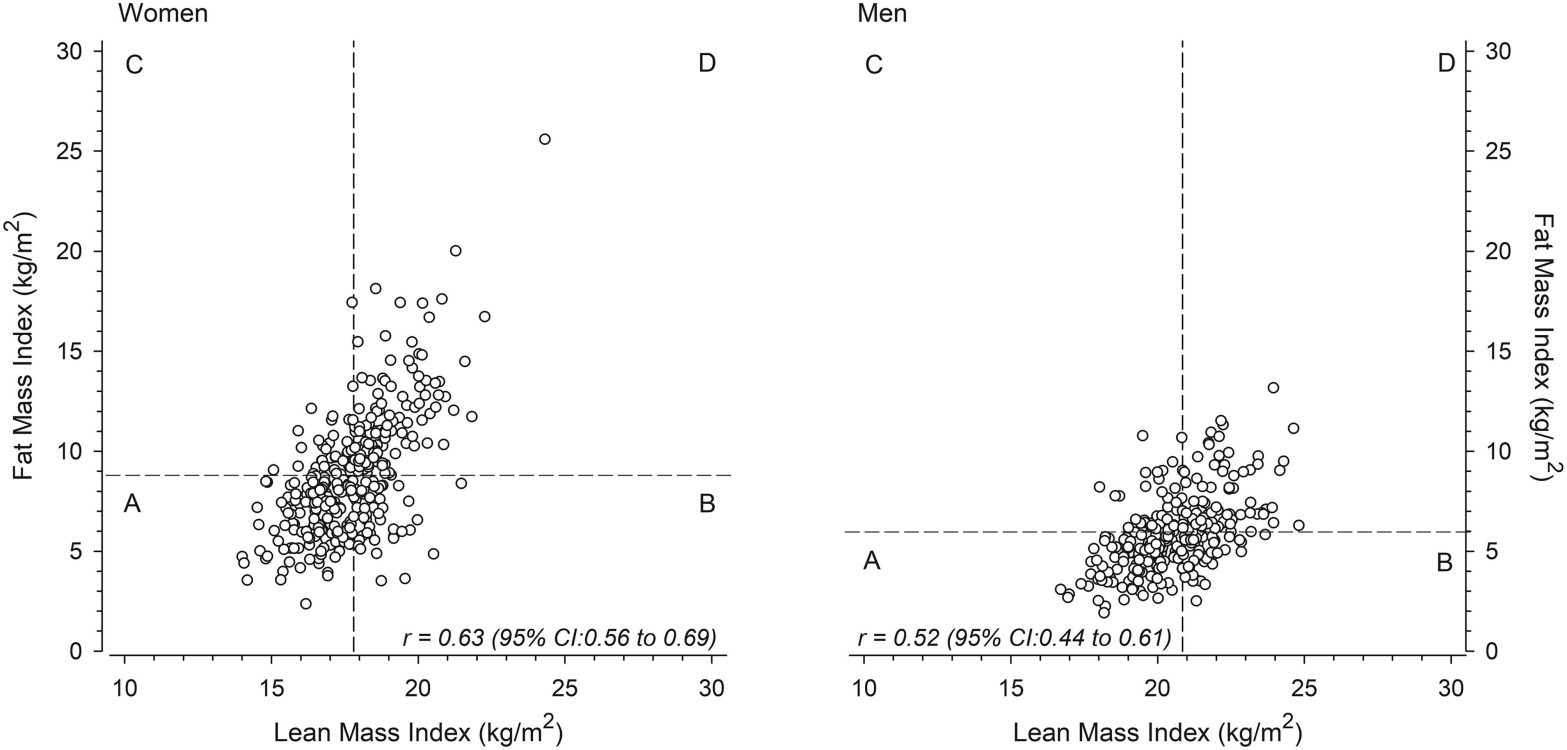
Mean lean mass index-fat mass index scatter plots for women (left) and men (right) divided into four groups (A, B, C, D) around the sex-specific median.

**Figure 2:**
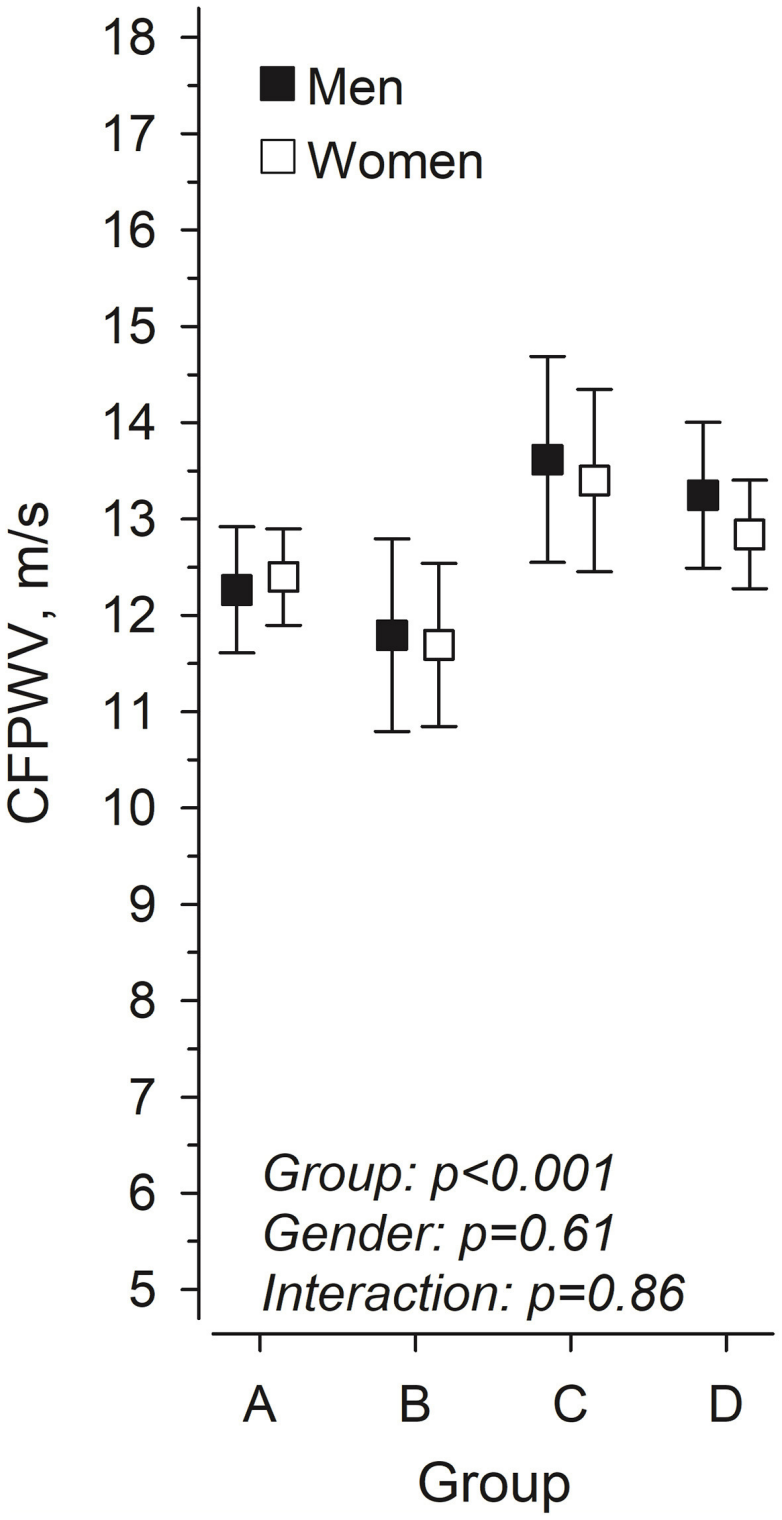
Pulse wave velocity in women and men at mean age 76 years according to body composition at 61 years, in four groups based on lean mass index (LMI) and fat mass index (FMI). (A) low LMI and low FMI, (B) high LMI and low FMI, (C) low LMI and high FMI, and (D) high LMI and high FMI.

Adiposity-related variables showed a strong association with PWV in the univariate analyses are therefore focused upon in greater details. The relationships between LMI and FMI and the median-split categories of body composition in women and men are shown in Figure 1. Figure 2 shows the crude unadjusted differences between the groups in relation to PVW. There is a statistically significant difference between the groups, with lower PWV values in groups A and B (both low FMI) compared to groups C and D (both high FMI). No sex differences were observed, nor any interactions between group and sex.

Table 2 shows linear regression models predicting PWV at follow-up based upon the four body composition categories in women and men combined and separately in women and men. Group B (high LMI and low FMI) was set as the reference group in further analyses. There were statistically significant differences between Group B (reference group) and Group C (low LMI and high FMI) and Group D (high LMI and high FMI) when women and men were combined. The trends and the beta values were similar also when women and men were analyzed separately. There was no significant difference between Group A (low LMI and low FMI) and Group B suggesting that a higher lean mass index was not associated with a better PWV in the presence of a low fat mass index. In a similar way there were no statistically significant differences between Groups C and D suggesting that a higher lean mass index was not associated with better vascular health in the presence of a high fat mass index.

**Table 2:** Regression models predicting pulse wave velocity 15 years later based upon body composition groups in men and women combined and separately in women and men.

	**Model I β (95% CI)**	**Model II β (95% CI)**	**Model III β (95% CI)**
**ALL**
B	Reference	Reference	Reference
A	0.09 (−0.02–0.20), *p* = 0.12	0.08 (−0.02–0.19), *p* = 0.15	0.08 (−0.03–0.19), *p* = 0.18
C	0.18 (0.08–0.28), *p* < 0.001	0.17 (0.07–0.27), *p* < 0.001	0.15 (0.05–0.24), *p* = 0.002
D	0.18 (0.07–0.29), *p* < 0.001	0.17 (0.06–0.28), *p* = 0.002	0.12 (0.01–0.23), *p* = 0.028
**Women**
B	Reference	Reference	Reference
A	0.11 (−0.04–0.27), *p* = 0.15	0.11 (−0.05–0.26), *p* = 0.18	0.11 (−0.04–0.26), *p* = 0.15
C	0.18 (0.05–0.31), *p* = 0.007	0.15 (0.02–0.28), *p* = 0.021	0.13 (0.01–0.26), *p* = 0.042
D	0.18 (0.02–0.33), *p* = 0.024	0.16 (0.01–0.31), *p* = 0.038	0.10 (−0.05–0.25), *p* = 0.18
**Men**
B	Reference	Reference	Reference
A	0.06 (−0.10–0.23), *p* = 0.43	0.06 (−0.10–0.22), *p* = 0.49	0.04 (−0.12–0.21), *p* = 0.60
C	0.18 (0.04–0.32), *p* = 0.012	0.18 (0.04–0.32), *p* = 0.012	0.15 (0.01– to 0.30), *p* = 0.042
D	0.19 (0.03–0.34), *p* = 0.020	0.18 (0.02–0.34), *p* = 0.023	0.14 (−0.02–0.31), *p* = 0.081

*Model I, Crude. Model II, Adjusted for age, LTPA, smoking history. Model III, Model II + further adjustment for systolic blood pressure, pulse rate, fasting blood glucose and cholesterol. β, standardized regression coefficient; A, low lean mass index and low fat mass index; B, high lean mass index and low fat mass index; C, low lean mass index and high fat mass index; D, high lean mass index and high fat mass index*.

## Discussion

In this study we assessed predictors of vascular health in old individuals from a life course perspective. Early life factors, in this case birth weight and body mass index at age 7 years, did not predict vascular health in the ninth decade of life. As expected, blood pressure in late midlife was a strong predictor of PWV in old age among men and women. Further, this study showed sex differences in predictors of PWV. In men primarily systolic blood pressure, fasting plasma glucose and adiposity related factors were associated with PWV during a 15-year follow-up. Interestingly in women the same factors seemed to be of importance but also markers of inflammation and lipid metabolism were associated with PWV measured in old age. Further a novel finding was that adiposity-related factors were strong predictors of vascular health, something not fully encapsulated in body mass index, lean body mass or body fat percentage alone. A higher fat mass index was associated with worse vascular health and was not ameliorated by a higher lean mass index. Our findings stress the importance to study fat and lean body mass simultaneously because of their close interaction with each other.

It is well known that blood pressure and arterial stiffness are closely correlated, also shown in the present study (5, 8). Besides the well-known association between blood pressure and arterial stiffness several other factors have also been proposed to be associated with arterial stiffness. These include non-hemodynamic factors like chronic low-grade inflammation, dyslipidemia and an impairment in glucose regulation (9–11). Our findings support these previous reports, but we also report sex differences in factors predicting arterial stiffness. In men systolic blood pressure, dysglycemia and a higher body fat percentage were predictors of arterial stiffness, while in women also dyslipidemia and inflammation related factors were predicting arterial stiffness.

Since dyslipidemia, dysglycemia and a low-grade chronic inflammation are all closely associated with overweight and obesity we wanted to focus upon the importance of body composition from a holistic point, simultaneously taking lean and fat mass into account (25). It is often assumed that a high lean body mass could ameliorate the negative effects of a higher fat mass. In order to be able to study the impact of fat and lean mass on vascular health we divided the study participants into four groups according to sex-specific median values for fat and lean mass indices. The group with low fat mass index and high lean mass index was set as the reference group. This because we hypothesized that those with a higher lean body mass and lower fat mass would be those with the best vascular health. In the present study we show that a higher fat mass index was associated with worse vascular health, which was not ameliorated by a higher lean body mass. The present findings are supported by findings from the Southampton Women's survey in the UK reporting that most vascular measurements in children at 8 to 9 years of age were related to BMI, and fat and lean mass contributed differently and PWV was positively associated with fat mass index. The authors conclude that “differences in vascular structure and function in relation to BMI probably represent combinations of adverse effects of fat mass, adaptive effects of body size, and relatively protective effects of lean mass” (26).

Factors active in early life and influencing e.g., fetal growth and childhood growth have been associated with a large number of NCDs including hypertension and type 2 diabetes (14, 15). Consequently, these factors have been proposed to be associated with vascular aging. In fact, an Austrian study reported that being born preterm or SGA were associated with higher PWV in a cohort studied at a mean age of 16 years (27). However, in the present study with a follow-up into the ninth decade of life we could not find any association between early life factors and vascular health when simultaneously taking into account factors active in later life. We have previously shown that body mass index at birth is positively associated with adult lean and fat mass indices (28). Therefore, we studied whether the influence of fat mass index on vascular health was mediated by body size at birth. However, based on the mediation models (data not shown) this was not the case.

Our findings suggest that lean mass without simultaneously taking fat mass into account is not a good predictor of vascular health among older people. These findings are supported by previous studies focusing upon physical performance among elderly people (29, 30). We believe that fat mass is to a certain degree confounding lean mass because individuals with a high fat mass tend to also have higher lean mass than those with low fat mass. Therefore, it is challenging to use lean mass as a predictor of various health outcomes without simultaneously accounting for the influence of fat mass (31). Overweight and obesity are known to be associated with several adverse health outcomes including CVD and Type 2 diabetes. The underlying mechanisms could include similar factors as those identified in predicting vascular health in the present study. Obesity is associated with a systemic low-grade inflammation and insulin resistance which are also known to contribute to the development of atherosclerosis (32, 33).

Strengths of the present study include a longitudinal study design with a 15-year clinical follow-up time and a register-based follow-up throughout the life course. We made assessment of body composition instead of relying solely on BMI, which allowed us to study fat and lean mass separately as well as simultaneously. Measurement of vascular health was based on measurements of carotid-femoral pulse wave velocity which is a valid way to measure arterial stiffness and predictive of fatal and non-fatal CVD as well as overall mortality (34, 35).

This study has some limitations. Our design was longitudinal, but we had no information on the participants PWV at baseline and therefore, we were not able to assess the change in PWV over the follow-up. Bioelectrical impedance analysis was used to determine body composition. The use of dual-energy x-ray absorptiometry would have ensured better validity but between-day precision of InBody 3.0 has been reported to be 2.7% and when compared to fat-free mass assessed by DXA scan, percent root mean square error of InBody 3.0 was 6%, which can be considered acceptable (20). As typical in studies including older adults, there was a loss of participants in the follow-up. Our participants were Europeans and the results may therefore not be generalizable to other populations.

Our findings stress the importance to study body composition and fat and lean body mass simultaneously because of their close interaction with each other also in relation to vascular health.

## Data Availability Statement

The raw data supporting the conclusions of this article will be made available by the authors, without undue reservation.

## Ethics Statement

The studies involving human participants were reviewed and approved by the Coordinating Ethics Committee, Hospital District of Helsinki and Uusimaa. The patients/participants provided their written informed consent to participate in this study.

## Author Contributions

JE contributed to research study design and interpretation of results and writing of manuscript. HK contributed to statistical analyses. MS, HK, and NW contributed to clinical data curation. All authors contributed to critical reading of the manuscript and interpretation of the results and approved the final submitted version.

## Funding

The HBCS has been supported by grants from Finska Läkaresällskapet, the Finnish Special Governmental Subsidy for Health Sciences, Academy of Finland (127437, 129306, 130326, 134791, 263924, and 315690), Samfundet Folkhälsan, Liv och Hälsa, EU FP7 [Developmental Origins of Healthy Aging (DORIAN)] project number 278603, and EU H2020-PHC-2014-DynaHealth grant 633595 and EU Horizon 2020 Award 733206 LIFECYCLE (all for the Helsinki Birth Cohort Study), European Commission, Horizon2020 award 733280 RECAP), Foundation for Cardiovascular Research, Foundation for Diabetes Research, Foundation for Pediatric Research, and Novo Nordisk Foundation, Signe and Ane Gyllenberg Foundation.

## Conflict of Interest

The authors declare that the research was conducted in the absence of any commercial or financial relationships that could be construed as a potential conflict of interest.

## Publisher's Note

All claims expressed in this article are solely those of the authors and do not necessarily represent those of their affiliated organizations, or those of the publisher, the editors and the reviewers. Any product that may be evaluated in this article, or claim that may be made by its manufacturer, is not guaranteed or endorsed by the publisher.
